# Leukocytosis and alteration of hemoglobin level in patients with severe COVID‐19: Association of leukocytosis with mortality

**DOI:** 10.1002/hsr2.194

**Published:** 2020-10-14

**Authors:** Babak Sayad, Zeinab Mohseni Afshar, Feizollah Mansouri, Zohreh Rahimi

**Affiliations:** ^1^ Infectious Diseases Research Center Kermanshah University of Medical Sciences Kermanshah Iran; ^2^ Behavioral Disease Research Center Kermanshah University of Medical Sciences Kermanshah Iran; ^3^ Department of Clinical Biochemistry Kermanshah University of Medical Sciences Kermanshah Iran

Dear Editors

The severe acute respiratory syndrome coronavirus 2 (SARS‐CoV‐2) is responsible for coronavirus disease 2019 (COVID‐19). The clinical characteristics of COVID‐19 pandemic announced by the World Health Organization (WHO) in March 2020^1^ may be presented by a severe form of pneumonia in some patients (10%‐15%) which might progress toward acute respiratory distress syndrome, multi‐organ failure and death.^2^ The clinical laboratory provides important information related to the COVID‐19 diagnosis, prognosis, and response to therapy.^3^ In severely affected patients with COVID‐19, leukocytosis was more prevalent as it was observed in11.4% severe COVID‐19 patients compared to 4.8% in patients with mild to moderate disease.^3^

For the first time we report white blood cells (WBCs) count and hemoglobin (Hb) level among patients with severe COVID‐19 from Iran (Table 1). The Farabi Hospital of Kermanshah University of Medical Sciences, Kermanshah, Iran as the second referral center of COVID‐19 in Kermanshah Province, with a population around 2 000 000, started the admission of COVID‐19 patients from March 7, 2020. A total number of 3574 individuals referred to the emergency unit of this hospital from March 7 to May 12, 2020 that among them 537 individuals were hospitalized due to COVID‐19 infection according to the chest CT scan and/or real time polymerase chain reaction. Seventy‐four severely affected patients (44 males and 30 females) were admitted to the intensive care unit (ICU) of the hospital. The mean age of all ICU admitted patients was 65.1 ± 17.1 years (23‐90 years), with the mean age of 61.4 ± 18.5 years (23‐90 years) in men and the mean age of 70.5 ± 13.2 years (39‐89 years) in women (Table 1).

**Table 1:** Age, hemoglobin (Hb) level, and white blood cells (WBCs) count in severe patients with COVID‐19

Variable	All patients n = 74	Survivors (n = 35) Mean ± SD	Non‐survivors (n = 39) Mean ± SD, *P*	Patients did not require intubation n = 48 Mean ± SD	Patients required intubation n = 26 Mean ± SD, *P*	Patients without comorbidities (n = 23) Mean ± SD	Patients with comorbidities (n = 51) Mean ± SD, *P*
Age (Years) Males (n = 44) Female (n = 30)	65.1 ± 17.1 (23–90) 61.4 ± 18.5 (23‐90) 70.5 ± 13.2 (39–89)	63.6 ± 18.7 58.2 ± 19.4 71.8 ± 14.6	66.5 ± 15.5, 0.48 64.4 ± 17.5, 0.27 69.3 ± 12.4, 0.62	63.7 ± 18.8 58.5 ± 19.4 73 ± 13.8	67.9 ± 12.8, 0.25 68.8 ± 13.9, 0.06 67.2 ± 12.3, 0.23	55.2 ± 16 52.8 ± 15 61.8 ± 18	69.4 ± 15.8, 0.001 66.5 ± 18.7, 0.012 72.6 ± 11.3, 0.2
WBCs ×10^3^/μL Males Females	9.1 ± 4.9 (1‐24.8) 8.9 ± 4.9 (1‐23.7) 9.4 ± 5 (1.6‐24.8)	7.6 ± 3.5 7.7 ± 4 7.3 ± 2.7	10.5 ± 5.6, 0.008 10.1 ± 5.4, 0.1 11.1 ± 5.9, 0.03	8.3 ± 4.1 8.6 ± 4.7 7.8 ± 2.6	10.5 ± 6, 0.098 9.7 ± 5.4, 0.51 11.3 ± 6.6, 0.09	8.2 ± 4.8 8.7 ± 5 6.7 ± 4.2	9.5 ± 4.9, 0.27 9.1 ± 4.9, 0.8 10 ± 5, 0.12
Hb (g/L) Males Females	140.2 ± 24.3 (75–205) 143.1 ± 23.5 (75‐205) 136.1 ± 25.3 (98‐200)	139.3 ± 22.9 141.4 ± 21.1 136.4 ± 25.9	141.1 ± 25.8, 0.75 144.8 ± 25.9, 0.63 135.9 ± 25.8, 0.96	141.8 ± 25.1 145.6 ± 24.6 135 ± 25	137.3 ± 23, 0.44 137.2 ± 20.3, 0.25 137.5 ± 26, 0.79	137.6 ± 19.3 142 ± 16.4 125.3 ± 23.1	141.4 ± 26.4, 0.49 143.8 ± 27.3, 0.78 138.8 ± 25.6, 0.24

Among all severely affected patients the mean WBCs count was 9.1 ± 4.9×10^9^/L (8.9 ± 4.9 × 10^9^/L in males and 9.4 ± 5 × 10^9^/L in females). In survivors the WBCs count was 7.6 ± 3.5 × 10^9^/L compared to 10.5 ± 5.6 × 10^9^/L (*P* = .008) in non‐survivors. Comparing survivors with non‐survivors indicated among non‐survivors 16 out of 39 patients (41%) had WBCs count upper normal range (>11 × 10^9^/L). However, only 4 out of 35 (11.4%) survived patients had WBCs count more than 11 × 10^9^/L (*χ* ^2^ = 8.2, *P* = .004, OR = 1.16 (CI 1.03‐1.3, *P* = .015). Also, the WBCs count was 10.5 ± 6 × 10^9^/L in patients required intubation compared to 8.3 ± 4.1 × 10^9^/L (*P* = .098) in those patients did not require intubation. Furthermore, higher WBCs count was detected in patients with comorbidities (hypertension, diabetes mellitus, coronary artery disease, cancer, renal transplantation, chronic obstructive pulmonary disease, and osteomyelitis) than those without comorbidities (9.5 ± 4.9 vs 8.2 ± 4.8 × 10^9^/L, *P* = 0.27; Table 1). There were seven patients with leukopenia (1‐3.8 × 10^9^/L) that four of them (57.1%) died.

The mean level of Hb was 140.2 ± 24.3 g/L (75‐205 g/L) in all patients. In men the mean level of Hb was 143.1 ± 23.5 g/L (75‐205 g/L) and in women was 136.1 ± 25.3 g/L (98‐200 g/L). The mean Hb level in 35 survived patients was 139.3 ± 22.9, and in 39 non‐survived patients was 141.1 ± 25.8 g/L (*P* = .75). There were 51 patients (68.9%) with comorbidities and the mean Hb level of 141.4 ± 26.4 g/L compared to 23 patients (31.1%) without comorbidities and the Hb level 137.6 ± 19.3 g/L (*P* = .49). Among patients 26 out of 74 (35.1%) required tracheal intubation with the mean Hb level 137.3 ± 23 g/L compared to the mean level of 141.8 ± 25.1 g/L in 48 patients who did not require intubation (*P* = .44; Table 1). There were 12 patients with low level of Hb (75‐115 g/L) that 5 of them (41.7%) died. Thirteen (59.1%) out of 22 patients with high levels of Hb (150‐205 g/L) died. Among studied patients only there was a severely anemic man (Hb 75 g/L) who required tracheal intubation that died after 3 days hospitalization. Among 40 patients with the normal Hb levels (116‐148 g/L) the rate of death was 52.5% (21 out of 40). There were 50 patients <60 years with WBCs count 8.8 ± 4.7 × 10^9^/L compared to 9.1 ± 4.9 × 10^9^/L in patients ≥60 years (*P* = .81). The Hb level in patients <60 years was 148 ± 22.6 g/L compared to 136.8 ± 24.7 g/L in patients ≥60 years (*P* = .059).

Related to the alteration of Hb level in patients infected with SARS‐CoV‐2 there are few studies that were subjected a meta‐analysis. In three out of four studies, the Hb level in severe COVID‐19 patients was significantly lower than those with milder forms. The meta‐analysis, included 1210 COVID‐19 patients that among them 224 were with severe disease (18.5%), suggested that in severe COVID‐19 patients compared to milder forms of the disease, Hb levels essentially decreased that needs clinical studies to investigate the effectiveness of blood transfusion support in prevention of progress to severe disease and death.^2^ Also, in a study consisted of nine severe ICU admitted Chinese patients with COVID‐19 the lower level of Hb [112 g/L (106‐130.5 g/L)] was detected compared to 18 severe non‐ICU patients [134.5 g/L (114.8‐144 g/L)] (*P* = .054).^4^ However, an artificial intelligence framework that has been developed according to dataset from 53 hospitalized patients (case series) in two hospitals of China predicted the clinical severity of COVID‐19 and later development of ARSD in patients with mild initial presentations. In their model, higher Hb levels were associated with poorer outcomes. According to this predictive model mild increased alanine aminotransferase, the presence of myalgias, and elevated hemoglobin level are the most predictive features with an accuracy of 70% to 80% in predicting the severity of disease and development of ARSD.^5^

Among patients with COVID‐19 from Kermanshah Province the mortality rate was grater in individuals with higher Hb levels. However, the level of Hb was lower in patients required tracheal intubation compared to patients did not require intubation.

In summary, our study indicated a significant association between leukocytosis and the rate of mortality in patients with COVID‐19. Also, our findings indicated the higher rate of mortality among COVID‐19 patients with higher Hb level. However, lower Hb level was detected in patients required tracheal intubation that its mechanism needs to be elucidated.

## CONFLICT OF INTEREST

The authors declare they have no conflict of interest.

## AUTHOR CONTRIBUTIONS

Visualization, Writing‐Review and Editing: Babak Sayad

Methodology and Formal Analysis: Zeinab Mohseni Afshar

Investigation and Editing: Feizollah Mansouri

Validation and Writing‐Original Draft Preparation: Zohreh Rahimi

       All authors have read and approved the final version of the manuscript.

       Zohreh Rahimi (corresponding author) had full access to all of the data in this study and takes complete responsibility for the integrity of the data and the accuracy of the data analysis.

### ETHICS STATEMENT

Although our study was an observational study, verbal consent was obtained from patients or from the next‐of‐kin. The Ethics Committee of Kermanshah University of Medical Sciences approved the study (Ethics code: IR.KUMS.REC.1399.049) and the study was in accordance with the principles of the Declaration of Helsinki II.

## Data Availability

The datasets used and analyzed during the current study are available from the corresponding author on reasonable request.
